# Laparoscopic entry techniques: Which should you prefer?

**DOI:** 10.1002/ijgo.14412

**Published:** 2022-09-01

**Authors:** Diego Raimondo, Antonio Raffone, Antonio Travaglino, Stefano Ferla, Manuela Maletta, Giulia Rovero, Federica Renzulli, Umberto de Laurentiis, Giulia Borghese, Marco Ambrosio, Paolo Salucci, Paolo Casadio, Antonio Mollo, Renato Seracchioli

**Affiliations:** ^1^ Division of Gynecology and Human Reproduction Physiopathology IRCCS Azienda Ospedaliero‐Universitaria di Bologna Bologna Italy; ^2^ Department of Medical and Surgical Sciences (DIMEC) University of Bologna Bologna Italy; ^3^ Pathology Unit, Department of Advanced Biomedical Sciences, School of Medicine University of Naples Federico II Naples Italy; ^4^ Gynecology and Obstetrics Unit, Department of Medicine, Surgery and Dentistry “Schola Medica Salernitana,” University of Salerno Baronissi Italy

**Keywords:** access, complications, guidelines, laparoscopy, minimally invasive, recommendation, safety, surgery

## Abstract

**Background:**

Despite a debate spanning two decades, no consensus has been achieved about the safest laparoscopic entry technique.

**Objectives:**

To update the evidence about the safety of the main different laparoscopic entry techniques.

**Search Strategy:**

Six electronic databases were searched from inception to February 2021.

**Selection Criteria:**

All randomized controlled trials (RCTs) comparing different laparoscopic entry techniques were included.

**Data Collection and Analysis:**

Entry‐related complications and total time for entry were compared among the different methods of entry calculating pooled odds ratios (ORs) and mean differences, with 95% confidence intervals (CIs); *P* < 0.05 was considered significant.

**Main Results:**

In total, 25 RCTs (6950 patients) were included. Complications considered were vascular, visceral and omental injury, failed entry, extraperitoneal insufflation, bleeding and infection at the trocar site bleeding, and incisional hernia. Compared to direct trocar, the OR for Veress needle was significantly higher for omental injury (OR 3.65, *P* < 0.001), for failed entry (OR 4.19, *P* < 0.001), and for extraperitoneal insufflation (OR 5.29, *P* < 0.001). Compared to the open method, the OR for Veress needle was significantly higher for omental injury (OR 4.93, *P* = 0.001), for failed entry (OR 2.99, *P* < 0.001), for extraperitoneal insufflation (OR 4.77; *P* = 0.04), and for incisional hernia. Compared to the open method, the OR for direct trocar was significantly lower for visceral injury (OR 0.17, *P* = 0.002) and for trocar site infection (OR 0.27, *P* = 0.001).

**Conclusions:**

The direct trocar method may be preferred over Veress needle and open methods as a laparoscopic entry technique since it appears associated to a lower risk of complications.

## Synopsis

The direct trocar method may be preferred over the Veress needle and open methods as a laparoscopic entry technique, due to a lower risk of complications.

## 1 INTRODUCTION

Surgery is by nature invasive and inevitably associated with trauma. Technical and technologic attempts have been made to minimize invasiveness, postoperative pain, and time to return to normal activity.^1^, ^2^ Laparoscopy allows for minimally invasive intra‐abdominal access via less extensive incisions.^2^ Moreover, several studies have shown that the overall risk of complications after laparoscopic surgery is lower than with laparotomy.^3^, ^4^

However, despite the safety of laparoscopic techniques, inadvertent serious injuries to the bowel, bladder, and vascular structures can occur.^5^, ^6^ The incidence is 3–4 per 1000 procedures, and more than 50% are related to the initial entry into the abdomen during the primary trocar insertion.^7^, ^8^ Minor complications, such as postoperative infection, subcutaneous emphysema, extraperitoneal insufflation, and trocar site hernia, are also associated with laparoscopic entry.^9^ In fact, the complications at entry constitute the “Achilles' heel” of this procedure, mainly due to complications unrecognized at the time of the injury.^10^ In order to reduce the risk of these complications, several entry maneuvers have been developed.^11^ The most used ones include the non‐insufflated open (Hasson) method, the conventional closed entry method with Veress needle CO_2_ pre‐insufflation, and the optical entry methods.^12^, ^13^ However, despite a debate spanning two decades, no consensus has been achieved about the safest entry technique.

A previous Cochrane review of laparoscopic entry techniques failed to demonstrate any evidence of benefit in terms of safety of one technique over another.^14^ Furthermore, international guidelines do not recommend one entry method over others.^15^, ^16^, ^17^, ^18^ Thus, nowadays, the choice regarding location, equipment, and method of entry basically depends on the experience of the surgeon and the availability of resources.

Some randomized controlled trials (RCTs) have recently provided additional data in the field,^19^, ^20^, ^21^, ^22^, ^23^, ^24^ but updated, pooled estimates are lacking.

The objective of the present study was to update the evidence about the safety of the main different laparoscopic entry techniques.

## 2 MATERIALS AND METHODS

### 2.1 Study protocol

The present study followed an a priori designed study protocol, defining methods for each review stage. Two authors independently concluded each stage and disagreements were solved by discussion among all authors.

The study was reported according to the criteria outlined in the *Cochrane Handbook for Systematic Reviews of Interventions*.^25^

### 2.2 Search strategy and study selection

Six electronic databases were searched (i.e. MEDLINE, ClinicalTrials.gov, Google Scholar, Web of Science, Scopus, Cochrane Library) from inception of the database to February 2021 for all relevant studies. Several combinations of the following words were adopted during the searches: “laparoscop*”; “access”; “entry”; “technique”; “incision”; “Veress”; “Veres”; “Hasson”; “visual entry system”; “minimally invasive surgery”; “pneumoperitoneum”; “open”; “closed”; “direct vision”; “abdominal entry”; “ancillary port*”; and “radially expanding port system.” The reference lists of each eligible study were also searched. No language restriction was applied.

All RCTs comparing the Veress needle, direct trocar, and open methods as laparoscopic entry techniques were included. Observational studies and literature reviews were excluded.

### 2.3 Risk of bias within studies assessment

The risk of bias within studies was assessed using the criteria outlined in the *Cochrane Handbook for Systematic Reviews of Interventions*.^25^ Each included trial was assessed for seven domains related to risk of bias as follows: (1) random sequence generation; (2) allocation concealment; (3) blinding of participants and personnel; (4) blinding of outcome assessment; (5) incomplete outcome data; (6) selective reporting; and (7) other bias.^25^ The authors' judgments were categorized as “low risk,” “unclear risk,” and “high risk” of bias.

### 2.4 Data extraction

Data extraction was performed without modification of the original data.

Two‐by‐two contingency tables were built by considering two dichotomized variables, as follows:

- laparoscopic entry technique, alternatively dichotomized as “Veress needle” versus “direct trocar methods,” “Veress needle” versus “open method,” and “direct trocar” versus “open methods”;
- complications related to the laparoscopic access, dichotomized as “present” versus “absent.”

All complications related to laparoscopic access with extractable data about the comparisons among laparoscopic entry techniques in the included studies were considered.

When extractable, data were also extracted about the total time for entry as means ± standard deviation (SD). Total time for entry was defined as the time in seconds from skin incision to intra‐abdominal visualization via laparoscope. When reported in time units different from seconds, it was converted.

### 2.5 Data analysis

The risk of complications related to laparoscopic access was compared among the different entry methods (Veress needle, direct trocar, and open) using the Peto odds ratio (OR) as events were expected to be rare; *P* < 0.05 was considered significant.

The mean difference in total time for entry among the different entry methods was also calculated.

ORs and mean differences were calculated as individual and pooled estimates, and reported graphically on forest plots, with 95% confidence intervals (CI).

Statistical heterogeneity among studies was evaluated by adopting the inconsistency index *I* ^2^: heterogeneity was a priori considered insignificant for *I* ^2^ below 25%, low for *I* ^2^ below 50%, moderate for *I* ^2^ less than 75%, and high for *I* ^2^ at 75% and above, as previously described.^26^, ^27^, ^28^, ^29^, ^30^ The fixed effect model was adopted for all analyses due to the use of Peto OR as a test.

Review Manager version 5.4 (The Nordic Cochrane Centre, Copenhagen, Denmark; Cochrane Collaboration, 2014) was used as software for data analysis.

## 3 RESULTS

### 3.1 Study selection and patients' characteristics

A total of 10 718 studies were identified through the searches in the electronic databases. After duplicate removal, 3120 studies were obtained and 54 were retained after title and abstract screening. Of these full‐text screened studies, 25 were included in the qualitative and quantitative analysis^13^, ^19^, ^20^, ^21^, ^22^, ^23^, ^24^, ^31^, ^32^, ^33^, ^34^, ^35^, ^36^, ^37^, ^38^, ^39^, ^40^, ^41^, ^42^, ^43^, ^44^, ^45^, ^46^, ^47^, ^48^ (Figure S1).

In total, 13 RCTs compared the Veress needle (*n* = 1995) and direct trocar method (*n* = 1926), 11 compared the Veress needle (*n* = 882) and open method (*n* = 907), and six compared the direct trocar (*n* = 936) and open methods (*n* = 955), with a total of 6950 patients included in the present analysis (Table S1).

The mean age and body mass index (BMI, calculated as weight in kilograms divided by the square of height in meters) of patients were in the range of 26 ± 4 to 52.5 ± 2.7 years and 21 ± 1.7 to 45.8 ± 5.9, respectively (Table S1).

### 3.2 Risk of bias within studies

Most of the included studies were judged as “low risk” of bias in most of the seven Cochrane domains related to the risk of bias. In particular, an unclear risk of bias was found in 12 studies^13^, ^21^, ^22^, ^24^, ^26^, ^28^, ^31^, ^32^, ^33^, ^35^, ^38^, ^40^ for the “Random sequence generation,” 18 studies^20^, ^21^, ^22^, ^23^, ^24^, ^29^, ^30^, ^31^, ^32^, ^33^, ^34^, ^35^, ^36^, ^37^, ^38^, ^39^, ^40^, ^42^ for “Allocation concealment,” 19 studies^15^, ^20^, ^22^, ^23^, ^24^, ^31^, ^32^, ^33^, ^35^, ^36^, ^37^, ^38^, ^42^, ^43^, ^44^, ^45^, ^46^, ^47^, ^48^ for “Blinding,” two studies^31^, ^33^ for “incomplete outcomes data,” and five studies^21^, ^24^, ^46^, ^47^, ^48^ for “Selective reporting” domains. On the other hand, only two studies were judged at high risk of bias: one^40^ in the “Blinding” domain, and the other^37^ in the “Selective reporting” domain (Figure S2).

Details about the authors' judgments are reported in the Supplementary Material.

### 3.3 Data analysis

Complications related to the laparoscopic access with extractable data from the included studies were as follows: vascular injury; visceral injury; omental injury; failed entry; extraperitoneal insufflation; bleeding at the trocar site; infection at the trocar site; and incisional hernia.

#### The Veress needle method versus the direct trocar method

**FIGURE 1:**
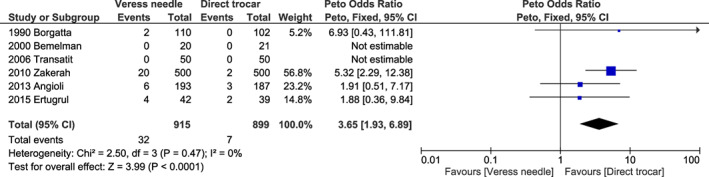
Forest plot reporting individual and pooled Peto odds ratios with 95% CIs for omental injury using a Veress needle compared to a direct trocar as the laparoscopic entry technique. CI, confidence interval.

**FIGURE 2:**
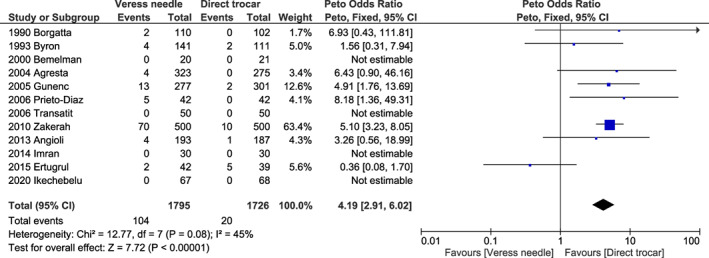
Forest plot reporting individual and pooled Peto odds ratios with 95% CIs for failed entry using a Veress needle compared to a direct trocar as the laparoscopic entry technique. CI, confidence interval.

**FIGURE 3:**
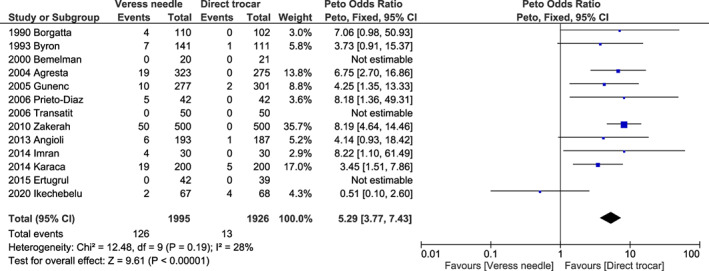
Forest plot reporting individual and pooled Peto odds ratios with 95% CIs for extraperitoneal insufflation using a Veress needle compared to a direct trocar as the laparoscopic entry technique. CI, confidence interval.

Compared to the direct trocar method, the Veress needle method showed an OR of 1.93 (95% CI 0.61–6.07, *I* ^2^ = 62%, *P* = 0.260) (Figure S3) for vascular injury; 1.21 (95% CI 0.27–5.37, *I* ^2^ = 55%, *P* = 0.800) (Figure S4) for visceral injury; 3.65 (95% CI 1.93–6.89, *I* ^2^ = 0%; *P* < 0.001) (Figure 1) for omental injury; 4.19 (95% CI 2.91–6.02, *I* ^2^ = 45%, *P* < 0.001) (Figure 2) for failed entry; 5.29 (95% CI 3.77–7.43, *I* ^2^ = 62%, *P* < 0.001) (Figure 3) for extraperitoneal insufflation; and 1.10 (95% CI 0.44–2.74, *I* ^2^ = 28%, *P* = 0.830) (Figure S5) for infection at the trocar site.

**FIGURE 4:**
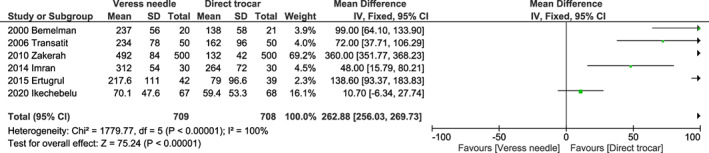
Forest plot of mean difference with 95% CIs in total time for entry between a Veress needle and direct trocar as the laparoscopic entry technique. CI, confidence interval.

The Veress needle method showed a higher total time for entry than the direct trocar method, with a mean difference of 262.88 s (95% CI 256.03–269.73, *I* ^2^ = 100%, *P* < 0.001) (Figure 4).

#### The Veress needle method versus the open method

**FIGURE 5:**
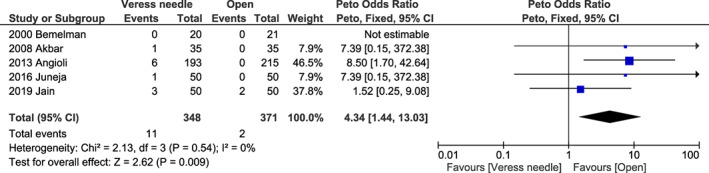
Forest plot reporting individual and pooled Peto odds ratios with 95% CIs for omental injury using a Veress needle compared to the open technique as the laparoscopic entry technique. CI, confidence interval.

**FIGURE 6:**
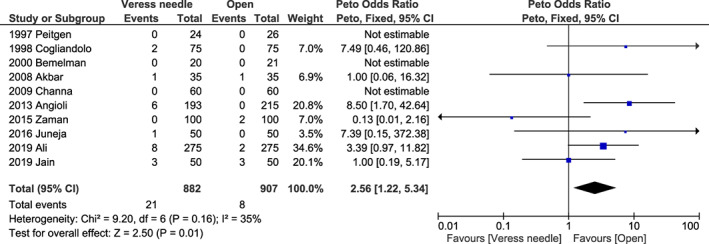
Forest plot reporting individual and pooled Peto odds ratios with 95% CIs for extraperitoneal insufflation using a Veress needle compared to the open technique as the laparoscopic entry technique. CI, confidence interval.

**FIGURE 7:**
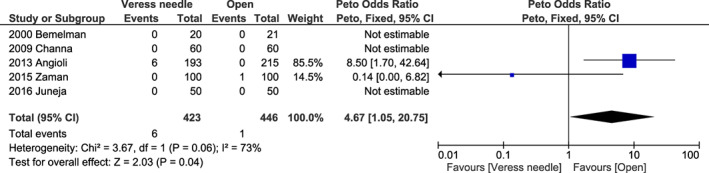
Forest plot reporting individual and pooled Peto odds ratios with 95% CIs for incisional hernia using a Veress needle compared to the open technique as the laparoscopic entry technique. CI, confidence interval.

Compared to the open method, the Veress needle method showed an OR of 1.96 (95% CI 0.53–7.30, *I* ^2^ = 0%, *P* = 0.310) (Figure S6) for vascular injury; 1.98 (95% CI 0.53–7.36, *I* ^2^ = 0%, *P* = 0.310) (Figure S7) for visceral injury; 4.34 (95% CI 1.44–13.03, *I* ^2^ = 0%, *P* = 0.009) (Figure 5) for omental injury; 1.40 (95% CI 0.69–2.81, *I* ^2^ = 78%, *P* = 0.350) (Figure S8) for failed entry; 2.56 (95% CI 1.22–5.34, *I* ^2^ = 35%, *P* = 0.010) (Figure 6) for extraperitoneal insufflation; 0.95 (95% CI 0.49–1.86, *I* ^2^ = 52%, *P* = 0.890) (Figure S9) for trocar site bleeding; 0.79 (95% CI 0.38–1.64, *I* ^2^ = 0%, *P* = 0.530) (Figure S10) for infection at the trocar site; and 4.77 (95% CI 1.05–20.75, *I* ^2^ = 73%, *P* = 0.040) (Figure 7) for incisional hernia.

#### The direct trocar method versus the open method

**FIGURE 8:**
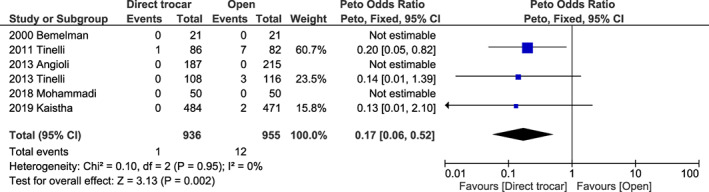
Forest plot reporting individual and pooled Peto odds ratios with 95% CIs for visceral injury using a direct trocar compared to the open technique as the laparoscopic entry technique. CI, confidence interval.

**FIGURE 9:**
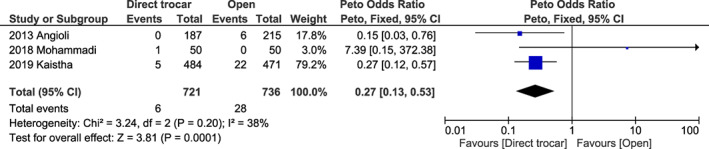
Forest plot reporting individual and pooled Peto odds ratios with 95% CIs for trocar site infection using a direct trocar compared to the open technique as the laparoscopic entry technique. CI, confidence interval.

Compared to the open method, the direct trocar method showed an OR of 0.31 (95% CI 0.05–1.83, *I* ^2^ = 68%, *P* = 0.200) (Figure S11) for vascular injury; 0.17 (95% CI 0.06–0.52, *I* ^2^ = 0%, *P* = 0.002) (Figure 8) for visceral injury; 8.68 (95% CI 0.89–84.34, *I* ^2^ not applicable, *P* = 0.060) (Figure S12) for omental injury; 1.63 (95% CI 0.61–4.38, *I* ^2^ = 0%, *P* = 0.330) (Figure S13) for failed entry; 7.39 (95% CI 0.15–372.38, *I* ^2^ not applicable, *P* = 0.320) (Figure S14) for extraperitoneal insufflation; 0.64 (95% CI 0.25–1.62, *I* ^2^ = 30%, *P* = 0.340) (Figure S15) for trocar site bleeding; and 0.27 (95% CI 0.13–0.53, *I* ^2^ = 38%, *P* = 0.001) (Figure 9) for infection at the trocar site.

**FIGURE 10:**
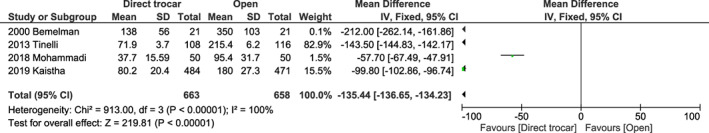
Forest plot of mean difference with 95% CIs in total time for entry between a direct trocar and the open technique as the laparoscopic entry technique. CI, confidence interval.

The direct trocar method showed a lower total time for entry than the open method, with a mean difference of −135.44 s (95% CI –136.65 to −134.23, *I* ^2^ = 100%, *P* < 0.001) (Figure 10).

## 4 DISCUSSION

The present study shows that the direct trocar method is associated with better outcomes than the Veress needle and open methods for laparoscopic entry. In particular, the direct trocar method is associated with a significantly lower risk of omental injury, failed entry, and extraperitoneal insufflation compared to the Veress needle method, and of visceral injury and infection at the trocar site compared to the open method. On the other hand, the Veress needle method is associated with a significantly higher risk of omental injury, extraperitoneal insufflation, and incisional hernia compared to the open method. Moreover, direct trocar was the fastest method, while the open method was the slowest one.

Which laparoscopic entry technique is preferable has been an unresolved issue for about 20 years. The issue has its roots in the attempt to identify the technique least associated with complications. Indeed, 50% of minor and major complications are related to the initial entry into the abdomen during the primary trocar insertion.^7^, ^8^, ^9^, ^10^, ^11^, ^12^, ^13^, ^14^ It was originally hypothesized that the Veress needle method would cause less major injury to intra‐abdominal structures, such as the bowel and vessels, because of the smaller diameter of the instrument.^14^ On the contrary, a 2019 Cochrane review of laparoscopic entry techniques concluded that there was no advantage of any technique in preventing the major complications of mortality, bowel or urinary injury, vascular injury, gas embolism, or injury to other organs.^14^

However, although conclusions may be affected by limitations related to included studies, such as poor overall quality and inappropriate statistical power to demonstrate a difference between these techniques when the incidence of major complications is so low, recommendations to drive the clinical practice and reduce complications on large numbers of patients are necessary.

In fact, to date, international guidelines do not recommend one entry method over the others^12^, ^15^, ^16^, ^17^, ^18^ and the choice is based on the preferences of the surgeon and the availability of resources. In this scenario, providing recommendations that only minimally reduce the risk of even few minor complications may represent an improvement.

The present analysis seems to indicate a preference for the direct trocar method over the Veress needle and open methods for laparoscopic entry, since it appears to be associated with a lower risk of omental injury, failed entry, and extraperitoneal insufflation compared to the Veress needle method, and of visceral injury and infection at the trocar site compared to the open method.

Although no difference in major complications was found, the findings might be enough to prefer the direct trocar method. Indeed, since the Veress needle is removed before primary trocar insertion, an omental needle injury can remain undetected for a prolonged period.^7^ On the other hand, due to extraperitoneal insufflations, beyond subcutaneous and omental emphysema, a gas embolism can also occur.^7^ In addition, the increased risk of failed entry seems to indicate a method that is more difficult to be learned and to be correctly completed. In fact, compared with the Veress needle technique, the direct trocar method reduces the number of blind steps from three to one. Thus, direct access with a trocar might have a positive impact on complication rates even higher in less experienced surgeons. Lastly, the direct trocar method appeared to be the best time‐sparing method.

Furthermore, the direct trocar method appeared to be preferred over the open method due to a decreased risk of visceral injury and infection at the trocar site. In fact, the open method mandatorily requires sharp surgical tools, while the direct trocar method consists of blunt insertion of the trocar. Moreover, the direct trocar method can benefit from the use of an optical trocar for a quicker visual identification of the bowel during the insertion of the trocar than the open method. Regarding the increased risk of infection at the site related to the open method, this might be due to the longer length of the procedure and the need to handle more surgical tools, which may facilitate the contamination of the surgical field.

The present study has some limitations. First, it was not possible to substratify the analysis based on several factors—such as the diameter of the trocars, angle or site of insertion of the Veress needle/trocar (umbilicus or not), type of trocar (optical or non‐optical), patient positioning, elevation of the abdominal wall using the surgeon's hand or forceps, and type of incision of the fascia in the open method (blunt dissection or sharp dissection)—which may affect the complication rate. Second, it was also impossible to substratify the analysis based on the characteristics of patients. In fact, laparoscopic entry may be more difficult and may show a higher rate of complications in the case of previous abdominal surgery or extremely high/low BMI.^11^, ^24^, ^31^, ^47^, ^48^ In future, it would be useful to assess the entry techniques in these categories of patients in an attempt to tailor the entry technique and further reduce the rate of complications. Third, data about the surgeons' experiences were not extractable for analysis; in fact, the volume and expertise of the surgeons could impact the outcomes of the methods of laparoscopic entry. Further studies are necessary to investigate these issues.

## 5 CONCLUSION

The direct trocar method may be preferred over the Veress needle and open methods as a laparoscopic entry technique, since it appears to be associated with a lower risk of omental injury, failed entry, and extraperitoneal insufflation compared to the Veress needle method, and of visceral injury and infection at the trocar site compared to the open method. Moreover, the direct trocar method appeared to be the best time‐sparing method.

Further studies are necessary to confirm these findings and to assess the laparoscopic entry technique to be preferred in patients at higher risk of complications related to laparoscopic entry, such as patients with previous abdominal surgery or extremely high/low BMI.

## AUTHOR CONTRIBUTIONS

DR: study conception, study design, study methods, data extraction, data analysis, and manuscript preparation; AR: study conception, study design, study methods, data analysis, manuscript preparation, and methods supervision; AT: study conception, study design, study methods, data extraction, data analysis, and manuscript preparation; SF: study design, study methods, data analysis, and manuscript preparation; MM: study conception, study design, study methods, data analysis, and manuscript preparation; GR: study conception, data extraction, data analysis, and manuscript preparation; FR: data extraction, data analysis, and manuscript preparation; GB: study design, data analysis, manuscript preparation, and methods supervision; UDL: methods supervision, study supervision, and manuscript revision; MA: study conception, study design, methods supervision, manuscript preparation, and whole study supervision; PS: methods supervision, study supervision, and manuscript revision; PC: study conception, study design, methods supervision, manuscript preparation, and whole study supervision; AM: study conception, study design, methods supervision, manuscript preparation, and whole study supervision; RS: study conception, study design, methods supervision, manuscript preparation, and whole study supervision. All authors approved of the final of the version to be published and agreed to be accountable for all aspects of the work in ensuring that questions related to the accuracy or integrity of any part of the work are appropriately investigated and resolved.

## CONFLICT OF INTEREST

The authors have no conflicts of interest.

## Supporting information


Appendix S1

## Data Availability

The data that support the findings of this study are available from the corresponding author upon reasonable request.
