# Supplementary material for: Laparoscopic entry techniques: Which should you prefer?
Source: Int J Gynaecol Obstet. 2022 Sep 1;160(3):742–50. doi: 10.1002/ijgo.14412 (PMC10087714; doi:10.1002/ijgo.14412)
Supplement: Supplementary file 1 — Appendix S1 [file IJGO-160-742-s001.zip › ijgo14412-sup-0001-FigureS1.pdf]

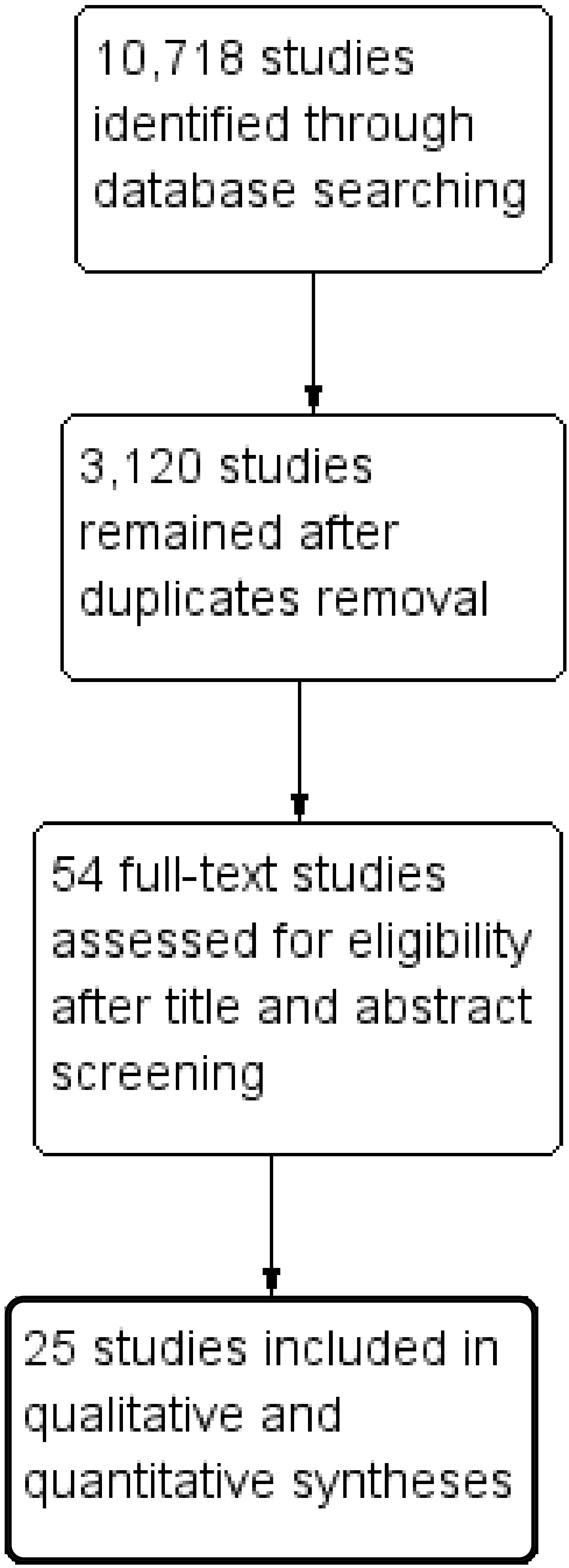
10,718 studies identified through database searching
3,120 studies remained after duplicates removal
54 full-text studies assessed for eligibility after title and abstract screening
25 studies included in qualitative and quantitative syntheses
