# Supplementary material for: Laparoscopic entry techniques: Which should you prefer?
Source: Int J Gynaecol Obstet. 2022 Sep 1;160(3):742–50. doi: 10.1002/ijgo.14412 (PMC10087714; doi:10.1002/ijgo.14412)
Supplement: Supplementary file 1 — Appendix S1 [file IJGO-160-742-s001.zip › ijgo14412-sup-0002-FigureS2A.pdf]

	Random sequence generation (selection bias)	Allocation concealment (selection bias)	Blinding of participants and personnel (performance bias)	Blinding of outcome assessment (detection bias)	Incomplete outcome data (attrition bias)	Selective reporting (reporting bias)	Other bias
1990 Borgatta	?	?	?	?	+	+	+
1993 Byron	+	?	+	+	+	+	+
1997 Peitgen	?	?	?	?	+	+	+
1998 Cogliandolo	+	?	?	?	+	+	+
2000 Bemelman	?	+	?	?	?	+	+
2004 Agresta	?	+	?	?	+	+	+
2005 Gunenc	+	?	+	+	+	+	+
2006 Prieto-Diaz	+	?	?	?	+	+	+
2006 Transatit	?	?	?	?	+	+	+
2008 Akbar	?	+	?	?	?	+	+
2009 Channa	+	?	+	+	+	+	+
2010 Zakerah	+	+	?	?	+	?	+
2011 Tinelli	+	?	?	?	+	?	+
2013 Angioli	+	+	?	?	+	+	+
2013 Tinelli	+	+	?	?	+	?	+
2014 Imran	+	?	?	?	+	+	+
2014 Karaca	?	?	?	?	+	+	+
2015 Ertugrul	?	?	−	−	+	+	+
2015 Zaman	?	?	?	?	+	−	+
2016 Juneja	?	?	+	+	+	−	+
2018 Mohammadi	?	?	?	?	+	−	+
2019 Ali	+	?	?	?	+	+	+
2019 Jain	+	?	?	?	+	+	+
2019 Kaistha	?	?	?	?	+	+	+
2020 Ikechebelu	+	+	+	+	+	+	+
