# Supplementary material for: Laparoscopic entry techniques: Which should you prefer?
Source: Int J Gynaecol Obstet. 2022 Sep 1;160(3):742–50. doi: 10.1002/ijgo.14412 (PMC10087714; doi:10.1002/ijgo.14412)
Supplement: Supplementary file 1 — Appendix S1 [file IJGO-160-742-s001.zip › ijgo14412-sup-0017-supinfo.docx]

**RISK OF BIAS WITHIN STUDIES ASSESSMENT**

1990 BORGATTA

| **DOMAIN** | **JUDGEMENT** | **REASON** |
| --- | --- | --- |
| Random sequence generation (selection bias) | Unclear risk | Method of randomization not stated |
| Allocation concealment (selection bias) | Unclear risk | Not referred to within paper |
| Blinding (performance bias and detection bias) | Unclear risk | Not referred to withing paper |
| Incomplete outcome data (attrition bias) | Low risk |  |
| Selective reporting (reporting bias) | Low risk |  |
| Other bias | Low risk |  |

1993 BYRON

| **DOMAIN** | **JUDGEMENT** | **REASON** |
| --- | --- | --- |
| Random sequence generation (selection bias) | Low risk |  |
| Allocation concealment (selection bias) | Unclear risk | Not referred to within paper |
| Blinding (performance bias and detection bias) | Low risk |  |
| Incomplete outcome data (attrition bias) | Low risk |  |
| Selective reporting (reporting bias) | Low risk |  |
| Other bias | Low risk |  |

1997 PEITGEN

| **DOMAIN** | **JUDGEMENT** | **REASON** |
| --- | --- | --- |
| Random sequence generation (selection bias) | Unclear risk | Method of randomization not stated |
| Allocation concealment (selection bias) | Unclear risk | Not referred to within paper |
| Blinding (performance bias and detection bias). | Unclear risk | Not referred to withing paper |
| Incomplete outcome data (attrition bias) | Low risk |  |
| Selective reporting (reporting bias) | Low risk |  |
| Other bias | Low risk |  |

1998 COGLIANDOLO

| **DOMAIN** | **JUDGEMENT** | **REASON** |
| --- | --- | --- |
| Random sequence generation (selection bias) | Low risk |  |
| Allocation concealment (selection bias) | Unclear risk | No blinding mentioned within the study |
| Blinding (performance bias and detection bias) | Unclear risk | No mention of blinding of participants or assessors in the text |
| Incomplete outcome data (attrition bias) | Low risk |  |
| Selective reporting (reporting bias) | Low risk |  |
| Other bias | Low risk |  |

2000 BEMELMAN

| **DOMAIN** | **JUDGEMENT** | **REASON** |
| --- | --- | --- |
| Random sequence generation (selection bias) | Unclear risk | Method of randomization unclear |
| Allocation concealment (selection bias) | Low risk |  |
| Blinding (performance bias and detection bias) | Unclear risk | Not stated |
| Incomplete outcome data (attrition bias) | Unclear risk | Participants reported as lost to follow-up because of intraoperative complications, which required conversion to a different technique; therefore, an intention-to-treat analysis may have been of value  “Two patients were withdrawn from inclusion during surgery, and no time-motion analysis was performed” |
| Selective reporting (reporting bias) | Low risk |  |
| Other bias | Low risk |  |

2004 AGRESTA

| **DOMAIN** | **JUDGEMENT** | **REASON** |
| --- | --- | --- |
| Random sequence generation (selection bias) | Unclear risk | Method of randomization unclear |
| Allocation concealment (selection bias) | Low risk |  |
| Blinding (performance bias and detection bias) | Unclear risk | No reference made |
| Incomplete outcome data (attrition bias) | Low risk |  |
| Selective reporting (reporting bias) | Low risk |  |
| Other bias | Low risk |  |

GUNENC 2005

| **DOMAIN** | **JUDGEMENT** | **REASON** |
| --- | --- | --- |
| Random sequence generation (selection bias) | Low risk |  |
| Allocation concealment (selection bias) | Unclear risk | Allocation concealment not referred to within the paper |
| Blinding (performance bias and detection bias) | Low risk |  |
| Incomplete outcome data (attrition bias) All outcomes | Low risk |  |
| Selective reporting (reporting bias) | Low risk |  |
| Other bias | Low risk |  |

2006 PRIETO DIAZ CHAVEZ

| **DOMAIN** | **JUDGEMENT** | **REASON** |
| --- | --- | --- |
| Random sequence generation (selection bias) | Low risk |  |
| Allocation concealment (selection bias) | Unclear risk | No reference to allocation concealment was made within the text of the paper |
| Blinding (performance bias and detection bias) | Unclear risk | No mention of blinding. |
| Incomplete outcome data (attrition bias) | Low risk |  |
| Selective reporting (reporting bias) | Low risk |  |
| Other bias | Low risk |  |

2006 TANSATIT

| **DOMAIN** | **JUDGEMENT** | **REASON** |
| --- | --- | --- |
| Random sequence generation (selection bias) | Unclear risk | Stated unclearly |
| Allocation concealment (selection bias) | Unclear risk | “Allocated by simple randomization” |
| Blinding (performance bias and detection bias) | Unclear risk | No mention of blinding. |
| Incomplete outcome data (attrition bias) | Low risk |  |
| Selective reporting (reporting bias) | Low risk |  |
| Other bias | Low risk |  |

AKBAR 2008

| **DOMAIN** | **JUDGEMENT** | **REASON** |
| --- | --- | --- |
| Random sequence generation (selection bias) | Unclear risk | “Selected patients were randomised into group A and B using sealed envelopes containing questionnaire. Seventy ProForma, 35 for each group, were prepared and sealed in blank envelopes. Each envelope contained one out of these seventy ProForma. After informed consent, an envelope was randomly fetched and opened” |
| Allocation concealment (selection bias) | Low risk |  |
| Blinding (performance bias and detection bias) | Unclear risk | “All the patients were operated upon under general anaesthesia by the same anaesthesia team. The surgical team consisted of a surgeon (principal author), two assistants, and one scrub nurse” |
| Incomplete outcome data (attrition bias) | Unclear risk | No losses to follow-up or lost data reported. However, some participants excluded if operation was converted to open cholecystectomy - figures not given |
| Selective reporting (reporting bias) | Low risk |  |
| Other bias | Low risk |  |

2009 CHANNA

| **DOMAIN** | **JUDGEMENT** | **REASON** |
| --- | --- | --- |
| Random sequence generation (selection bias) | Low risk |  |
| Allocation concealment (selection bias) | Unclear risk | No mention of allocation concealment in the text. |
| Blinding (performance bias and detection bias) | Low risk |  |
| Incomplete outcome data (attrition bias) | Low risk |  |
| Selective reporting (reporting bias) | Low risk |  |
| Other bias | Low risk |  |

2010 ZAKERAH

| **DOMAIN** | **JUDGEMENT** | **REASON** |
| --- | --- | --- |
| Random sequence generation (selection bias) | Low risk |  |
| Allocation concealment (selection bias) | Low risk |  |
| Blinding (performance bias and detection bias) | Unclear risk | Blinding not mentioned |
| Incomplete outcome data (attrition bias) | Low risk |  |
| Selective reporting (reporting bias) | Unclear risk | All outcomes reported, but different statistical analysis used. |
| Other bias | Low risk |  |

2011 TINELLI

| **DOMAIN** | **JUDGEMENT** | **REASON** |
| --- | --- | --- |
| Random sequence generation (selection bias) | Low risk |  |
| Allocation concealment (selection bias) | Unclear risk | Method not stated |
| Blinding (performance bias and detection bias). | Unclear risk | Blinding not mentioned |
| Incomplete outcome data (attrition bias) | Low risk |  |
| Selective reporting (reporting bias) | Unclear risk | No data conversion. P values not stated for each outcome. No omission of outcomes and no subsets of data. |
| Other bias | Low risk |  |

2013 ANGIOLI

| **DOMAIN** | **JUDGEMENT** | **REASON** |
| --- | --- | --- |
| Random sequence generation (selection bias) | Low risk |  |
| Allocation concealment (selection bias) | Low risk |  |
| Blinding (performance bias and detection bias) | Unclear risk | Blinding was not mentioned in the study |
| Incomplete outcome data (attrition bias) | Low risk |  |
| Selective reporting (reporting bias) | Low risk |  |
| Other bias | Low risk |  |

2013 TINELLI

| **DOMAIN** | **JUDGEMENT** | **REASON** |
| --- | --- | --- |
| Random sequence generation (selection bias) | Low risk |  |
| Allocation concealment (selection bias) | Low risk |  |
| Blinding (performance bias and detection bias). | Unclear risk | Blinding not mentioned |
| Incomplete outcome data (attrition bias). | Low risk |  |
| Selective reporting (reporting bias) | Unclear risk | No data conversion. P values not stated for each outcome. No omission of outcomes and no subsets of data. |
| Other bias | Low risk | No other source of bias identified |

2014 IMRAN

| **DOMAIN** | **JUDGEMENT** | **REASON** |
| --- | --- | --- |
| Random sequence generation (selection bias) | Low risk |  |
| Allocation concealment (selection bias) | Unclear risk | No mention of allocation concealment in the text. |
| Blinding (performance bias and detection bias). | Unclear risk | No mention of blinding in the text. |
| Incomplete outcome data (attrition bias) | Low risk |  |
| Selective reporting (reporting bias) | Low risk |  |
| Other bias | Low risk |  |

2014 KARACA

| **DOMAIN** | **JUDGEMENT** | **REASON** |
| --- | --- | --- |
| Random sequence generation (selection bias) | Unclear risk | Method of randomization non described. |
| Allocation concealment (selection bias) | Unclear risk | No reference to allocation concealment was made within the text of the paper |
| Blinding (performance bias and detection bias) | Unclear risk | No mention of blinding of participants personnel, or outcome assessors |
| Incomplete outcome data (attrition bias) | Low risk |  |
| Selective reporting (reporting bias) | Low risk |  |
| Other bias | Low risk |  |

2015 ERTUGRUL

| **DOMAIN** | **JUDGEMENT** | **REASON** |
| --- | --- | --- |
| Random sequence generation (selection bias) | Unclear risk | “Patients were randomized with the sealed envelope technique” No method for random sequence generation provided in the text |
| Allocation concealment (selection bias) | Unclear risk | All patients were informed about the operation, and detailed consent forms were taken before surgery” Unclear whether participant consent was obtained after allocation. No information available as to whether envelopes were serially numbered or sealed shut |
| Blinding (performance bias and detection bias) | High risk | it was not a blinded study |
| Incomplete outcome data (attrition bias) | Low risk |  |
| Selective reporting (reporting bias) | Low risk |  |
| Other bias | Low risk |  |

2015 ZAMAN

| **DOMAIN** | **JUDGEMENT** | **REASON** |
| --- | --- | --- |
| Random sequence generation (selection bias) | Unclear risk | Described in the abstract as “prospective randomized double blind study” No reference to randomisation made in the main body of text. No method of random sequence generation provided |
| Allocation concealment (selection bias) | Unclear risk | Not clear whether participants consented before or after allocation. No attempt made to describe allocation concealment |
| Blinding (performance bias and detection bias). All outcomes. | Unclear risk | Study described in the abstract as “prospective randomized double blind study”. No mention of blinding in the main body of the paper |
| Incomplete outcome data (attrition bias) All outcomes | Low risk |  |
| Selective reporting (reporting bias) | High risk | Individual significance values not given for different complications. Overall significance for complication rates between 2 groups given as P < 0.05. |
| Other bias | Low risk |  |

2016 JUNEJA

| **DOMAIN** | **JUDGEMENT** | **REASON** |
| --- | --- | --- |
| Random sequence generation (selection bias) | Unclear risk | Method of randomization not explicitly stated “assigned randomly” |
| Allocation concealment (selection bias) | Unclear risk | No mention of allocation concealment in the text. |
| Blinding (performance bias and detection bias) | Low risk |  |
| Incomplete outcome data (attrition bias) | Low risk |  |
| Selective reporting (reporting bias) | Unclear risk | Data presented as numbers of cases and percentages; |
| Other bias | Low risk |  |

2018 MOHAMMADI

| **DOMAIN** | **JUDGEMENT** | **REASON** |
| --- | --- | --- |
| Random sequence generation (selection bias) | Unclear risk | Method of randomization non described. |
| Allocation concealment (selection bias) | Unclear risk | No reference to allocation concealment was made within the paper |
| Blinding (performance bias and detection bias) | Unclear risk | No mention of blinding of participants personnel, or outcome assessors |
| Incomplete outcome data (attrition bias) | Low risk |  |
| Selective reporting (reporting bias) | Unclear risk | Data presented as numbers of cases and percentages |
| Other bias | Low risk |  |

2019 ALI

| **DOMAIN** | **JUDGEMENT** | **REASON** |
| --- | --- | --- |
| Random sequence generation (selection bias) | Low risk |  |
| Allocation concealment (selection bias) | Unclear risk | No mention of allocation concealment in the text. |
| Blinding (performance bias and detection bias) | Unclear risk | Not referred to withing paper |
| Incomplete outcome data (attrition bias) | Low risk |  |
| Selective reporting (reporting bias) | Low risk |  |
| Other bias | Low risk |  |

2019 JAIN

| **DOMAIN** | **JUDGEMENT** | **REASON** |
| --- | --- | --- |
| Random sequence generation (selection bias) | Low risk |  |
| Allocation concealment (selection bias) | Unclear risk | No mention of allocation concealment in the text. |
| Blinding (performance bias and detection bias). | Unclear risk | No mention of blinding in the text. |
| Incomplete outcome data (attrition bias) | Low risk |  |
| Selective reporting (reporting bias) | Low risk |  |
| Other bias | Low risk |  |

2019 KAISTHA

| **DOMAIN** | **JUDGEMENT** | **REASON** |
| --- | --- | --- |
| Random sequence generation (selection bias) | Unclear risk | central randomization system with a simple convenient sampling method. |
| Allocation concealment (selection bias) | Unclear risk | No mention of allocation concealment in the text. |
| Blinding (performance bias and detection bias) | Unclear risk | No mention of blinding in the text. |
| Incomplete outcome data (attrition bias) | Low risk |  |
| Selective reporting (reporting bias) | Low risk |  |
| Other bias | Low risk |  |

2020 IKECHEBELU

| **DOMAIN** | **JUDGEMENT** | **REASON** |
| --- | --- | --- |
| Random sequence generation (selection bias) | Low risk |  |
| Allocation concealment (selection bias) | Low risk |  |
| Blinding (performance bias and detection bias) | Low risk |  |
| Incomplete outcome data (attrition bias) | Low risk |  |
| Selective reporting (reporting bias) | Low risk |  |
| Other bias | Low risk |  |
