# Supplementary material for: Laparoscopic entry techniques: Which should you prefer?
Source: Int J Gynaecol Obstet. 2022 Sep 1;160(3):742–50. doi: 10.1002/ijgo.14412 (PMC10087714; doi:10.1002/ijgo.14412)
Supplement: Supplementary file 1 — Appendix S1 [file IJGO-160-742-s001.zip › ijgo14412-sup-0018-supinfo.docx]

| YEAR | AUTHOR | COUNTRY | NO. OF SUBJECTS | AGE (years) | BMI (kg/m2) | GENDER  (%) | TYPE OF SURGERY |
| --- | --- | --- | --- | --- | --- | --- | --- |
| 1990 | Borgatta | USA | 212 | 36.4  35.6 | 27.3  26.3 | M(0)  W: 212 (100) | Gynaecologic |
| 1993 | Byron | USA | 252 | - | - | W: 252 (100) | Gynaecologic |
| 1997 | Peitgen | Germany | 50 | 52.5 ± 2.7 | - | M: 29 (58)  W: 21 (42) | General Surgery |
| 1998 | Cogliandolo | Italy | 150 | 53.7 ± 4.2  55.6 ± 3.7 | - | M: 44 (29)  W: 106 (71) | General Surgery |
| 2000 | Bemelman | Netherlands | 62 | 51.2 ± 18.7  45 ± 14.9  48.7 ± 12.7 | 25.7 ± 3.7  28 ± 12.5  25.6 ± 5.0 | M: 30 (50)  W: 30 (50) | General Surgery |
| 2004 | Agresta | Italy | 598 | 40.2 ± 17.8  41.1 ± 15.6 | 21.6 ± 4.4  21.1±5.3 | -  - | General Surgery |
| 2005 | Gunenc | Turkey | 578 | - | - | - | Gynaecologic |
| 2006 | Prieto D. C. | Mexico | 84 | 49 ± 17.6 | - | M: 14 (16,6)  W: 70 (83,4) | General Surgery |
| 2006 | Tansatit | Thailand | 100 | 33.4 ± 4.6  34.6 ± 4.8 | 21.3 ± 3.2 21.4 ± 3.3 | W:100 (100) | Gynaecologic |
| 2008 | Akbar | Pakistan | 81 | 42 | - | - | General Surgery |
| 2009 | Channa | Pakistan | 120 | 43 ± 7 | - | M: 13 (11)  W: 107 (89) | General Surgery |
| 2010 | Zakerah | Egypt | 1000 | 25.9 ± 4.2 25.7 ± 4.1 | 26.6 ± 3.1 26.5 ± 2.8 | M: 0 (0)  W: 1000 (100) | Gynaecologic |
| 2011 | Tinelli | Italy | 168 | 42.3 ± 2.5  41.8 ± 3.3 | 28.3 ± 6.2  27.9 ± 5.9 | M: 0 (0)  W: 168 (100) | Gynaecologic |
| 2013 | Angioli | Italy | 595 | 33.9 ± 10.3  36.1 ± 10.5  38.0 ± 12.0 | 25.1 ± 5.6  22.8 ± 6.0  26.03 ± 2.6 | M: 0 (0)  W: 595 (100) | Gynaecologic |
| 2013 | Tinelli | Italy | 224 | 36.1 ± 4.5  35.7 ± 5.8 | 34.9 ± 5.1  35.1 ± 4.9 | M: 0 (0)  W: 224 (100) | Gynaecologic |
| 2014 | Imran | Pakistan | 60 | 40 ± 4 | - | M: 5 (8)  W: 55 (92) | General Surgery |
| 2014 | Karaca | Turkey | 400 | 46.8±9.5  48.1±10.4 | 28.3±2.7  28.2±3.1 | M:56 (14)  W:344 (86) | General Surgery |
| 2015 | Ertugrul | Turkey | 81 | 37.7 – 10.8  37.8 – 9.6 | 45.8 – 5.9  45.2 – 6.5 | M: 28 (34)  W: 53 (66) |  |
| 2015 | Zaman | India | 200 | - | - | - | General Surgery |
| 2016 | Juneja | India | 100 | 42±7 years | - | M: 43 (43)  W: 57 (57) | General Surgery |
| 2018 | Mohammadi | Iran | 100 | 41.4 ± 11.2  41.6 ± 15 | - | M: 51 (51%)  W: 49 (49%) | Urologic |
| 2019 | Ali | Pakistan | 550 | - | - | M: 137 (24.9)  W: 413 (75.1) | General Surgery |
| 2019 | Jain | India | 100 | - | - | M: 62 (62)  W: 38 (38) | General Surgery |
| 2019 | Kaistha | India | 950 | 42.8 ± 18.6  45.2 ± 21.1 | 23.7 ± 4.6  24.0 ± 3.9 | M: 260 (27)  W: 695 (73) | General Surgery |
| 2020 | Ikechebelu | Nigeria | 135 | 35.5 ± 4.3 35.6 ± 5.1 | 34.8 ± 4.3 33.9 ± 2.0 | M: 0 (0)  W: 135 (100) | Gynaecologic |
