# Supplementary figures and images for: Laparoscopic entry techniques: Which should you prefer?
Source: Int J Gynaecol Obstet. 2022 Sep 1;160(3):742–50. doi: 10.1002/ijgo.14412 (PMC10087714; doi:10.1002/ijgo.14412)

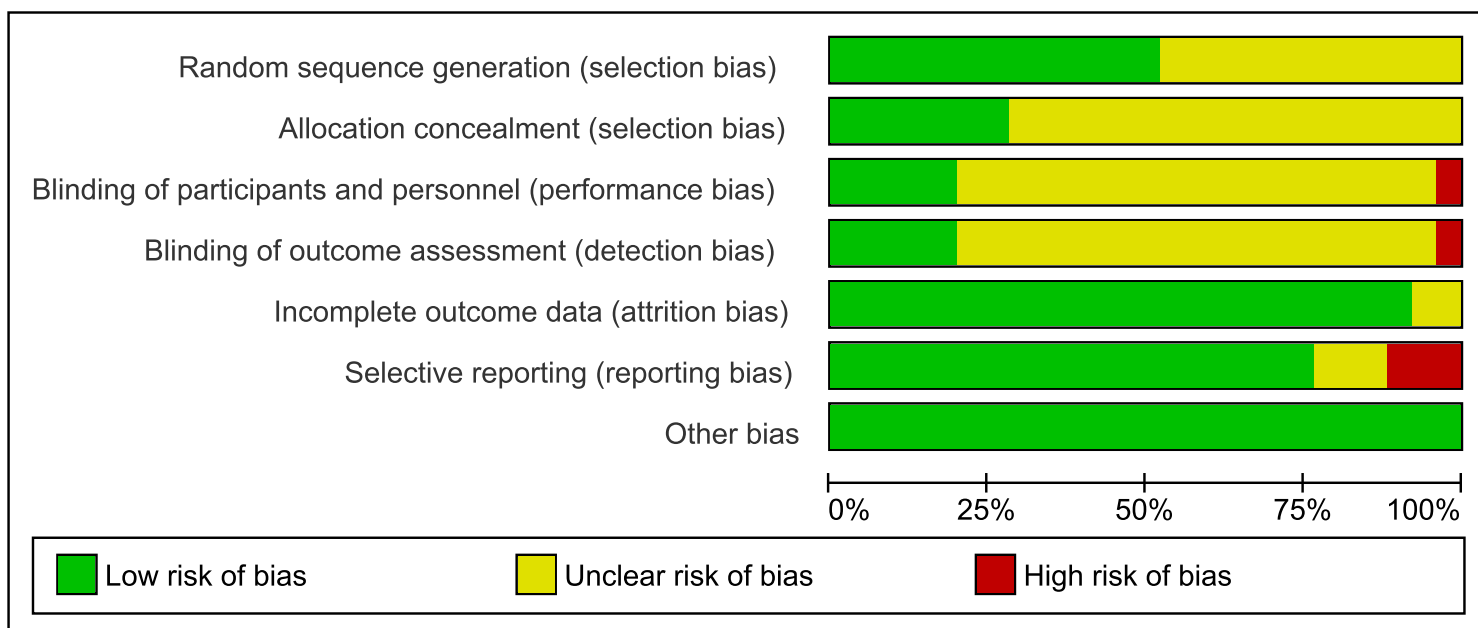

Supplement: Supplementary file 1 — Appendix S1 [file IJGO-160-742-s001.zip › ijgo14412-sup-0003-FigureS2B.pdf]

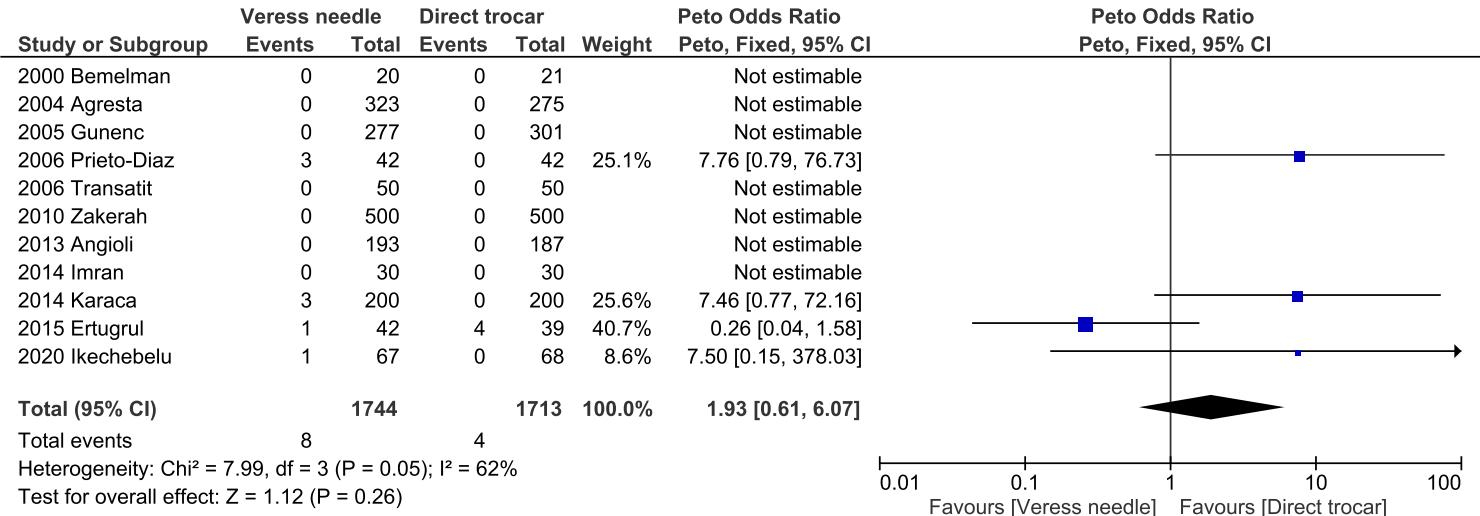

Supplement: Supplementary file 1 — Appendix S1 [file IJGO-160-742-s001.zip › ijgo14412-sup-0004-FigureS3.pdf]

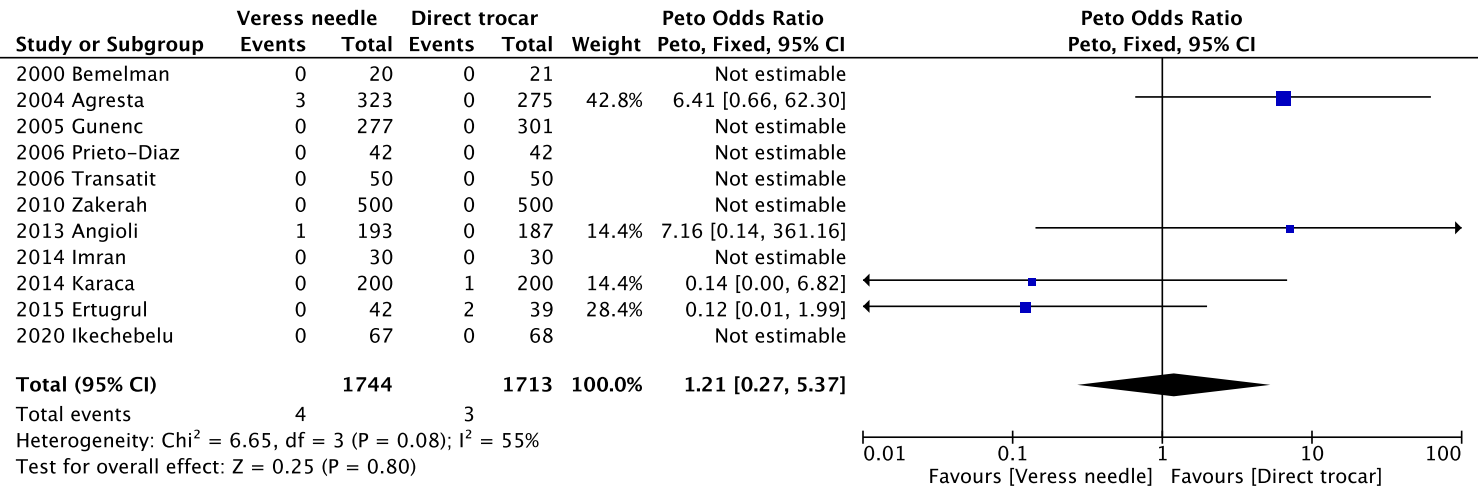

Supplement: Supplementary file 1 — Appendix S1 [file IJGO-160-742-s001.zip › ijgo14412-sup-0005-FigureS4.pdf]

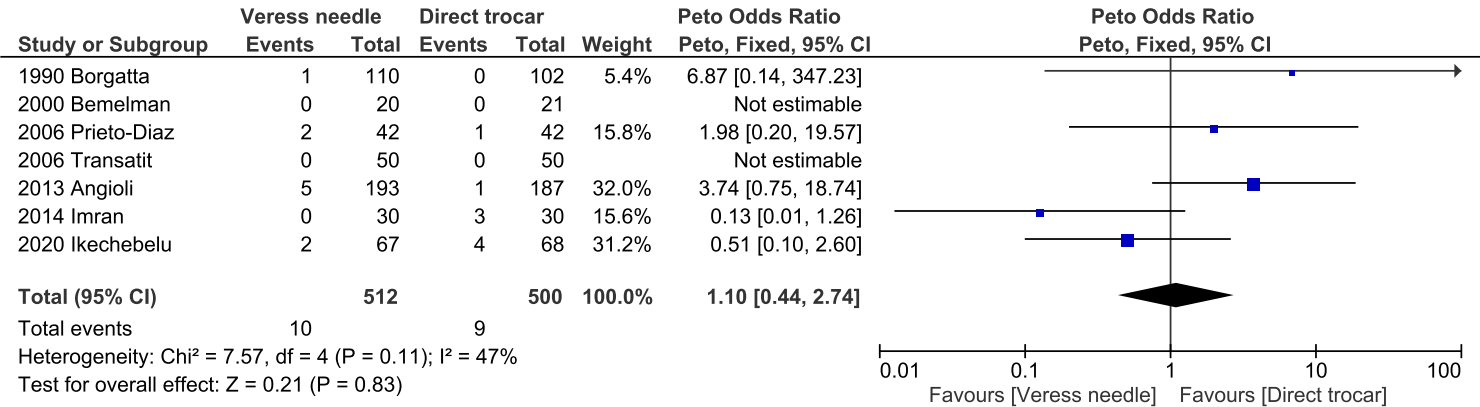

Supplement: Supplementary file 1 — Appendix S1 [file IJGO-160-742-s001.zip › ijgo14412-sup-0006-FigureS5.pdf]

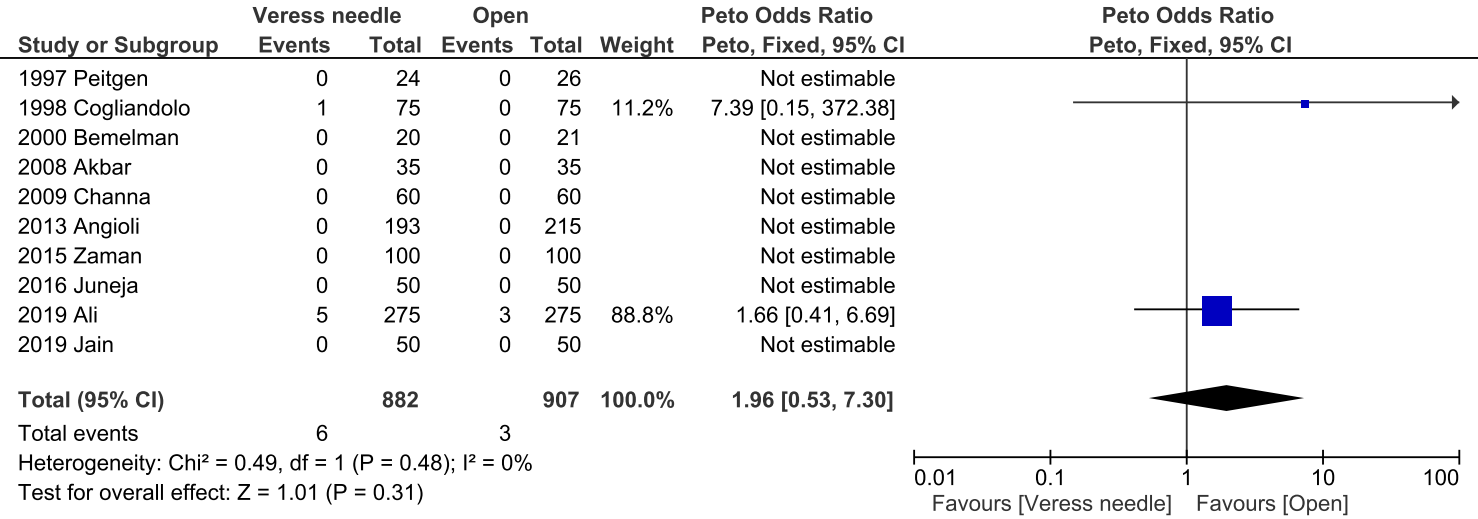

Supplement: Supplementary file 1 — Appendix S1 [file IJGO-160-742-s001.zip › ijgo14412-sup-0007-FigureS6.pdf]

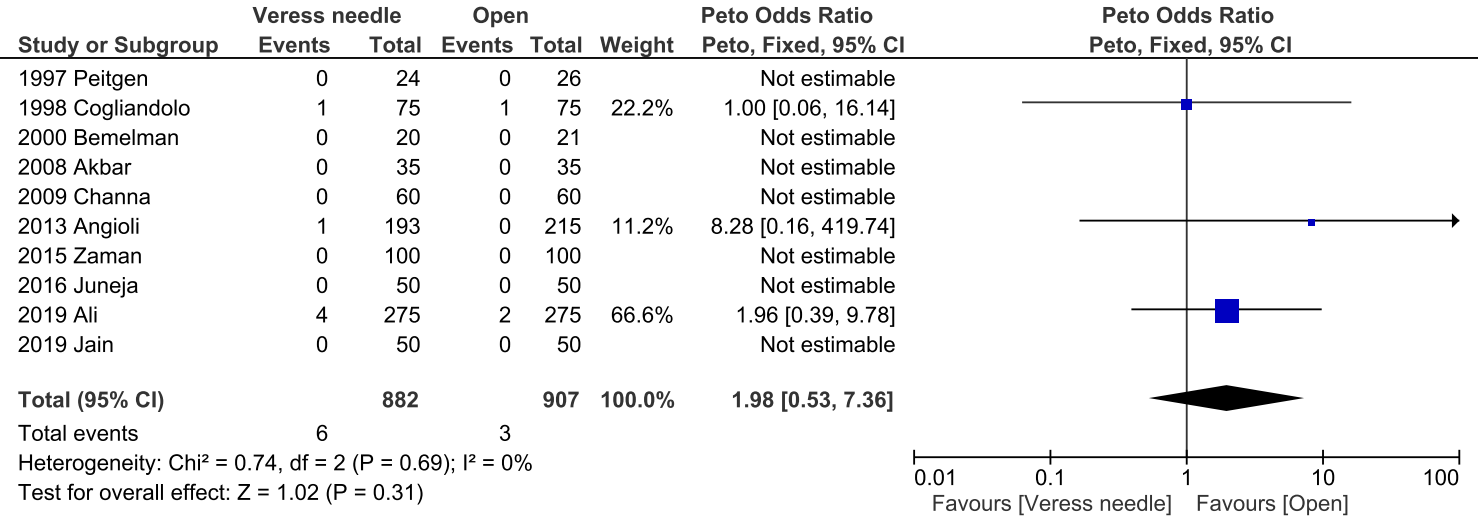

Supplement: Supplementary file 1 — Appendix S1 [file IJGO-160-742-s001.zip › ijgo14412-sup-0008-FigureS7.pdf]

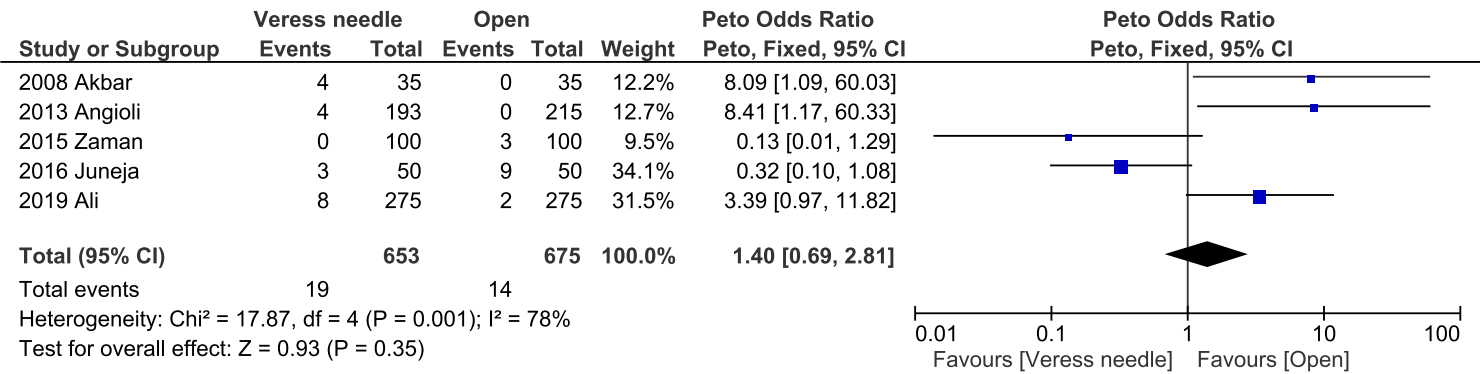

Supplement: Supplementary file 1 — Appendix S1 [file IJGO-160-742-s001.zip › ijgo14412-sup-0009-FigureS8.pdf]

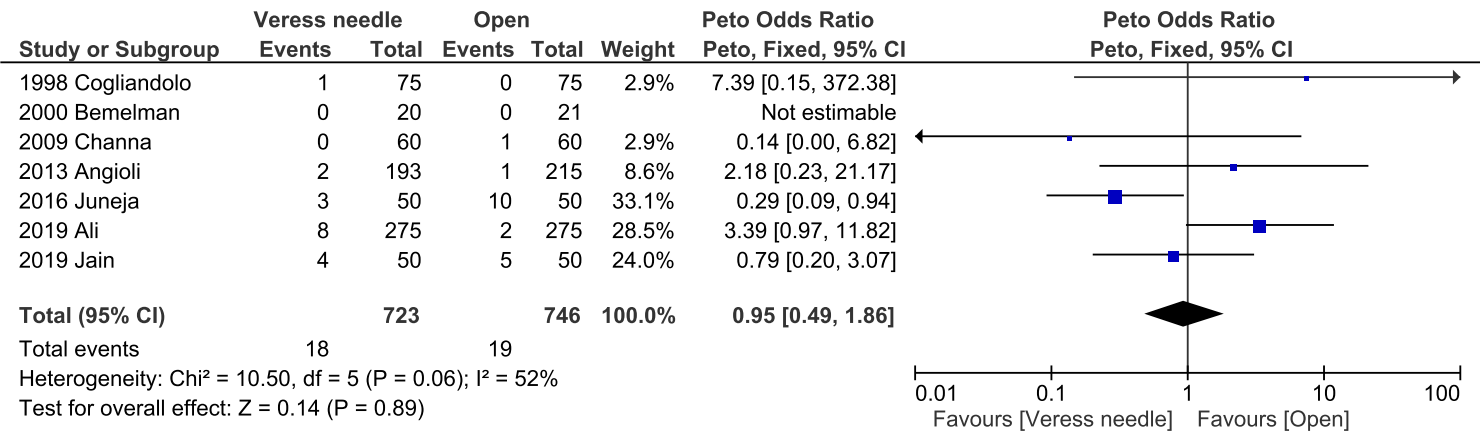

Supplement: Supplementary file 1 — Appendix S1 [file IJGO-160-742-s001.zip › ijgo14412-sup-0010-FigureS9.pdf]

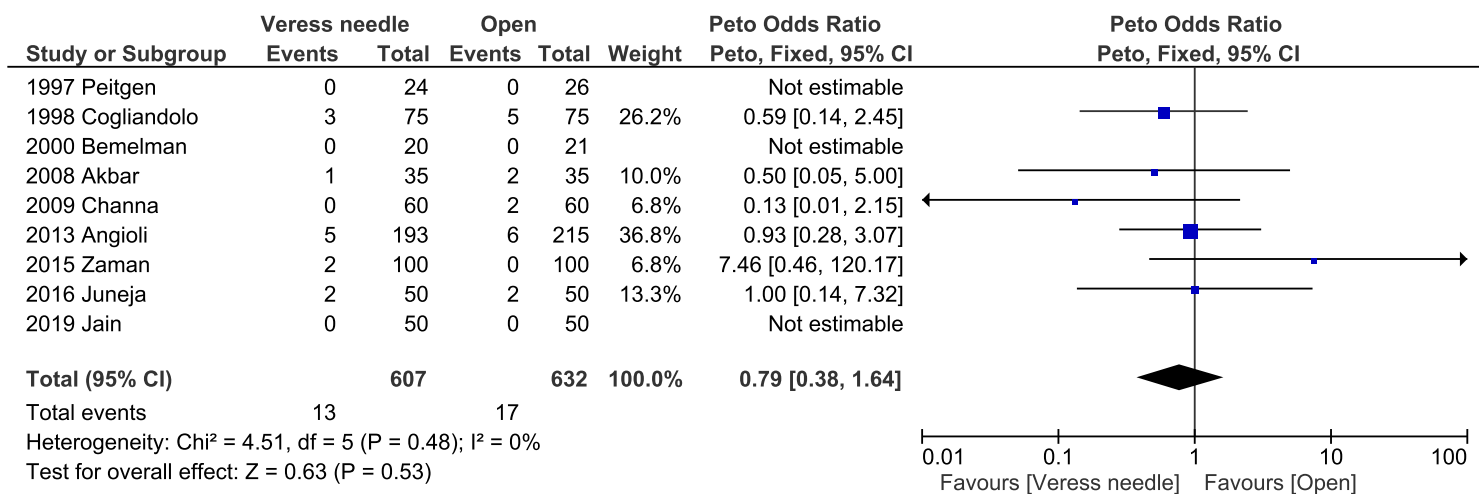

Supplement: Supplementary file 1 — Appendix S1 [file IJGO-160-742-s001.zip › ijgo14412-sup-0011-FigureS10.pdf]

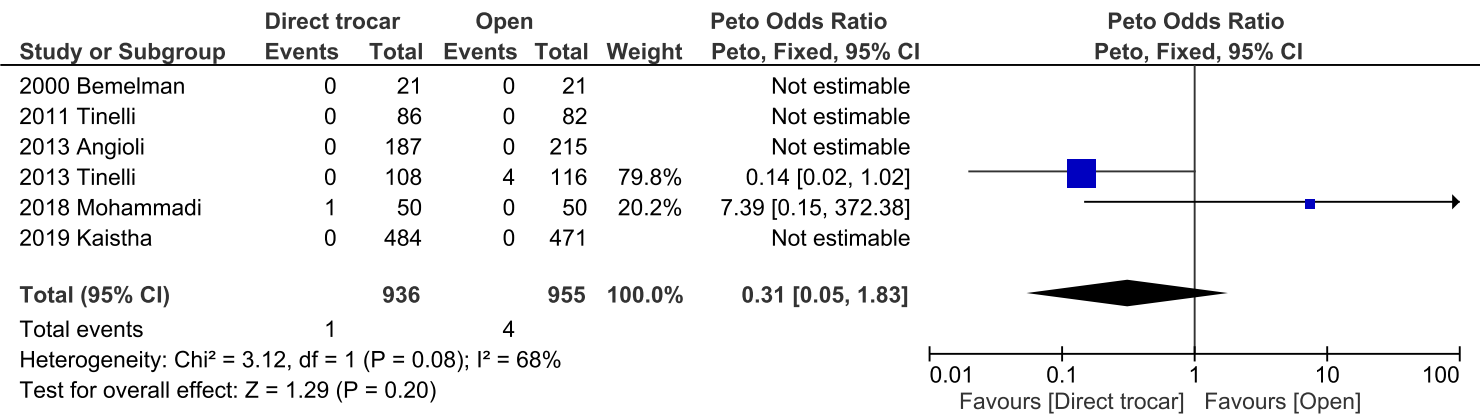

Supplement: Supplementary file 1 — Appendix S1 [file IJGO-160-742-s001.zip › ijgo14412-sup-0012-FigureS11.pdf]

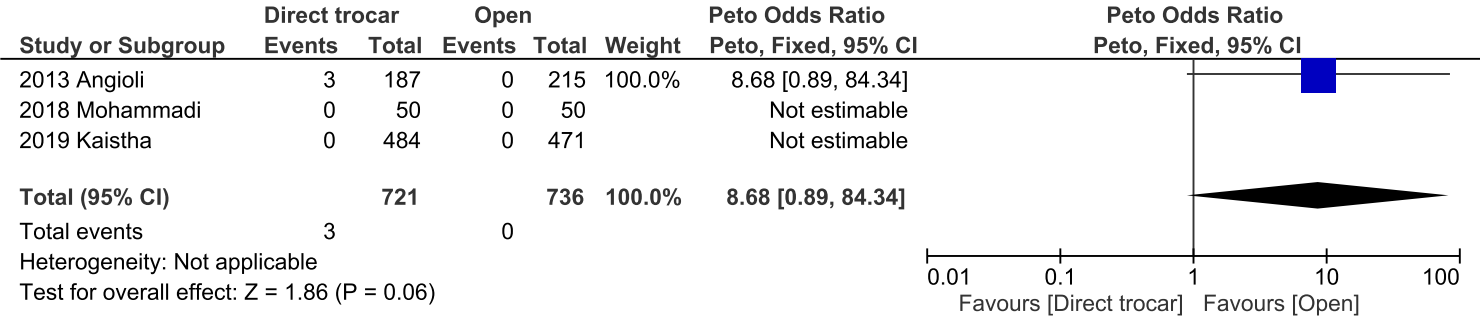

Supplement: Supplementary file 1 — Appendix S1 [file IJGO-160-742-s001.zip › ijgo14412-sup-0013-FigureS12.pdf]

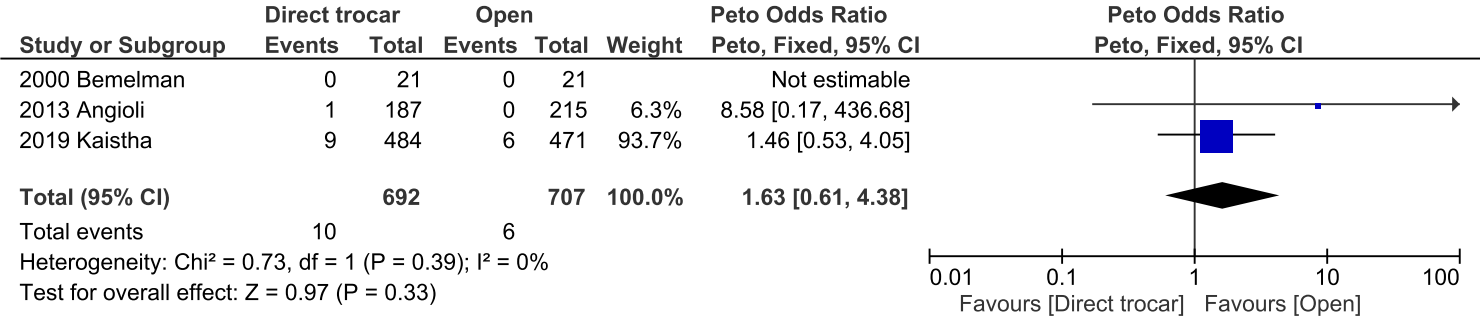

Supplement: Supplementary file 1 — Appendix S1 [file IJGO-160-742-s001.zip › ijgo14412-sup-0014-FigureS13.pdf]

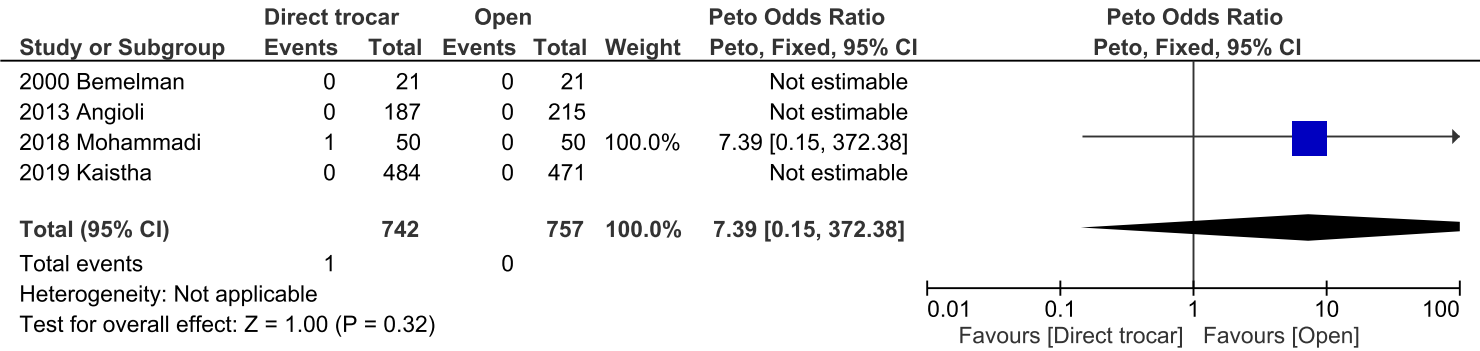

Supplement: Supplementary file 1 — Appendix S1 [file IJGO-160-742-s001.zip › ijgo14412-sup-0015-FigureS14.pdf]

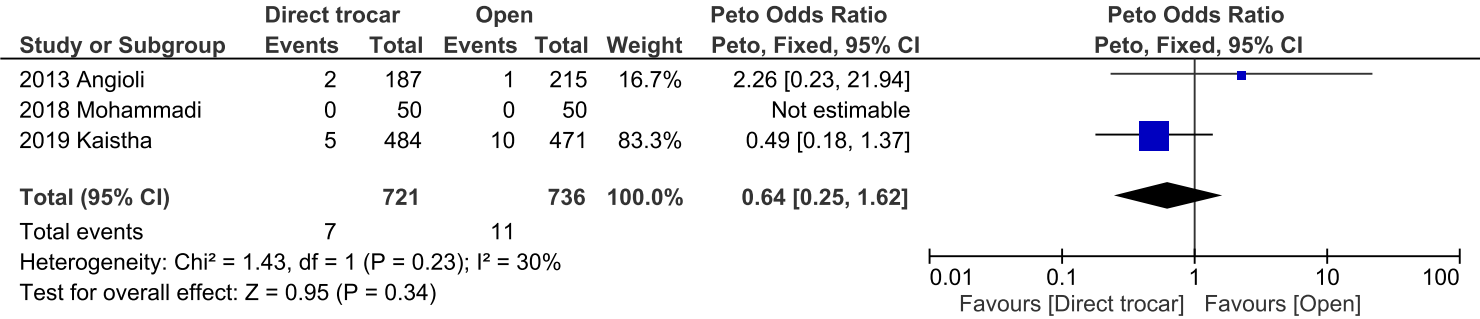

Supplement: Supplementary file 1 — Appendix S1 [file IJGO-160-742-s001.zip › ijgo14412-sup-0016-FigureS15.pdf]
